# Prediction of Creep Curves Based on Back Propagation Neural Networks for Superalloys

**DOI:** 10.3390/ma15196523

**Published:** 2022-09-20

**Authors:** Bohao Ma, Xitao Wang, Gang Xu, Jinwu Xu, Jinshan He

**Affiliations:** Collaborative Innovation Center of Steel Technology, University of Science and Technology Beijing, Beijing 100083, China

**Keywords:** metals and alloys, creep, artificial intelligence, machine learning, θ projection model

## Abstract

Creep deformation is one of the main failure forms for superalloys during service and predicting their creep life and curves is important to evaluate their safety. In this paper, we proposed a back propagation neural networks (BPNN) model to predict the creep curves of MarM247LC superalloy under different conditions. It was found that the prediction errors for the creep curves were within ±20% after using six creep curves for training. Compared with the θ projection model, the maximum error was reduced by 30%. In addition, it is validated that this method is applicable to the prediction of creep curves for other superalloys such as DZ125 and CMSX-4, indicating that the model has a wide range of applicability.

## 1. Introduction

Creep resistance is an important attribute for the long-term use of high temperature structural materials [1,2]. The long-term reliability of superalloy components in gas turbines must ensure that no excessive distortion occurs during service. Therefore, it is of great significance to predict the creep behavior of superalloys, especially the remnant creep life corresponding to a certain creep strain. In 1985, Evans and Wilshire proposed the θ projection method to predict the creep life of materials by describing the creep curves [3]. Specifically, this method describes the creep strain ε in function of time t as: ${\varepsilon =\theta }_{1}\left(1-\exp\left(-\theta_{2}t\right)\right){+\theta }_{3}(\exp\left(\theta_{4}t\right)-1)$, where θ_1_ and θ_3_ represent the amplitude of the primary and tertiary creep stages, respectively, while θ_2_ and θ_4_ represent the inflection of these two stages. Various numerical methods [4,5] have been presented to predict creep curves in recent years. Kulkarni et al. [4] proposed the Liu–Murakami creep damage model to calculate creep strain. The calculated results between this method and experimental data of 316 stainless at 600 °C were in less error. Li et al. [5] proposed a crystal plasticity finite element (CPFE) based model to predict the creep fatigue crack initiation life. The method was successfully applied to the prediction of a series of creep tests of GH4169 superalloy at 650 °C. In these methods, the θ projection method is widely used because of its simple principle. However, the secondary creep behavior is not clearly described, which takes the longest during creep. In addition, creep mechanisms would be various at different conditions which has important influence on the creep curves [6]. More importantly, the influence of the morphology change of the microstructure on creep curves cannot be reflected by these methods. Therefore, it is difficult to satisfy the accuracy requirements for creep curve prediction by phenomenological model construction.

With the development of computational science, artificial intelligence has gradually penetrated into the field of materials and data-driven machine learning has been widely used [7,8]. Back propagation neural network (BPNN), as a kind of artificial neural network, can deal with the regression task of complex nonlinear data by back propagation algorithm, which is low-cost and highly efficient [9]. Recently, BPNN has been successfully applied to the prediction of residual stress in FGH4095 superalloy after laser shock [10] and the prediction of tensile strength, yield strength and elongation of unvulcanized AISI 10xx series carbon steel [11]. In view of the extraordinary ability of BPNN for nonlinear-data mapping, it provides us with a new way to predict creep curves under different conditions from the perspective of creep curve data.

In the present study, we have proposed using a BPNN model to predict creep curves under different conditions. Datasets of six creep curves were used to train the model and the creep curves under another four creep conditions were applied to validate the model. In addition, the predicted results were compared to the experimental data and the prediction of the θ projection model. Moreover, the creep curve data of DZ125 and CMSX-4 superalloys in the literature [12,13] were also used to validate the model.

## 2. Data and Model Construction

### 2.1. Creep Data

**Figure 1:**
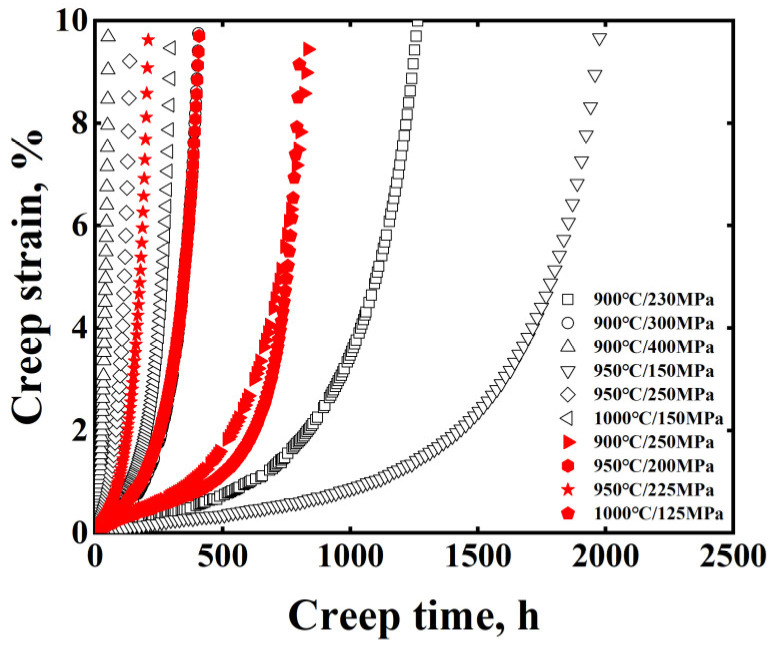
Creep curves obtained under different conditions for MarM247LC superalloy. The black curves were used for fitting the Larson–Miller parameter and training the model; the red ones were used to validate the model.

In this study, creep tests of MarM247LC superalloy were conducted. The obtained creep curves were shown in Figure 1. All creep curves consist of three stages: (1) the primary creep stage where the creep rate decreases with time, (2) the secondary creep stage where the creep rate is almost constant, (3) the tertiary creep stage where the creep rate increases rapidly with time leading to the failure. By comparing the creep curves at 900 °C, the creep life (*t_f_*) grows with the decreasing stress. Under these conditions, the primary and secondary stages are much longer at low stress when *t_f_* is longer. In addition, the *t_f_* shows a sharply decreasing trend at 250 MPa when the temperature increased from 900 °C to 950 °C. At the same time, the primary and secondary stages are much shorter at high temperature when *t_f_* is shorter. The above phenomena indicate that the shape of the creep curve is correlated with *t_f_*. On the other hand, by comparing the creep curves at 900 °C/250 MPa and 1000 °C/125 MPa, the creep curves are different even though the *t_f_* values at these two conditions are similar. In addition, the *t_f_* parameter reflects the degree of damage to the microstructure. This illustrates that creep curves are influenced by temperature and stress, apart from *t_f_*. Therefore, the temperature, stress and *t_f_* were taken as input parameters to predict the creep strain:

*ε* = *f* (*T*, *σ*, *t*, *t_f_*) (1)

where *ε* is the strain, *T* is the temperature, *σ* is the stress and *t* is the time. To get the input *t_f_* values for different creep conditions, the Larson–Miller method P_LM_(*σ*) = 10^−3^*T*(C_LM_ + lg*t_f_*) was taken.

### 2.2. Back Propagation Neural Network

BPNN is a quite efficient tool for computing data mapping. It establishes the connection between input data and output data by simulating the working process of biological neurons. The typical BPNN contains an input layer, one or more hidden layers and an output layer. The BPNN is trained by using the back propagation (BP) algorithm. During the learning process, the signal will propagate from the input layer to the output layer. The hidden layer contains a large number of neurons which can used to process the non-linear mapping between data. After the forward propagation of the signal, the gradient descent will be used to adjust the weights and biases during the backward propagation, so as to minimize the target error. The work of selecting the activation function and the number of neurons in each hidden layer is very complex because too few neurons in each hidden layer may not be able to sufficiently learn the characteristics of the data, and too many neurons will overfit the data. Usually, researchers need to use a trial and error procedure to settle this. In the present work, we use mean square error (MSE) as the objective function, set 400 iterations and stop training when the MSE of the validation dataset is less than 0.0001 to prevent overfitting. Meanwhile, the MSE obtained by partial BPNN structure is shown in Table 1. The results show that the MSE is the minimum when the number of hidden layers is three meets 16, 8 and 8, respectively. After much training, the parameters for the BPNN model are listed in Table 2.

The temperature, stress, time and strain are under different dimensions, which would reduce the convergence speed and accuracy within the neural network. To avoid such problems, all data were normalized into dimensionless units. By using the tanh activation function, the activation function is more sensitive when the data is between 0.1 and 0.9. This is because the derivative of tanh function in this interval is large, which directly affects the back propagation derivation process. In this process, the weight and bias are updated according to the results of derivation. The larger derivative is beneficial to the updating of parameters. Therefore, the input and output parameters were normalized within the range from 0.1 to 0.9 using the relation [14,15] given by Equation (2).

*y_n_* = 0.1 + 0.8(*y* − 0.95*y_min_*)/(1.05*y_max_* − 0.95*y_min_*) (2)

where *y_n_* is the normalized value, *y* is the experimental data, and *y_max_* and *y_min_* are the maximum and minimum values of *y*, respectively.

## 3. Results and Discussion

**Figure 2:**
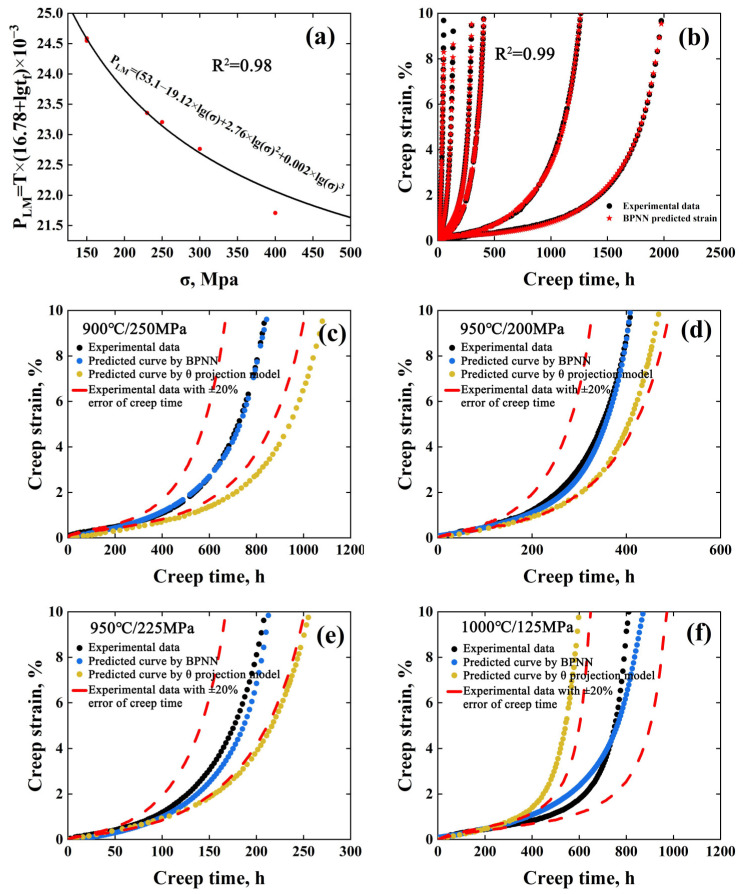
(**a**) The fitted result by Larson–Miller equation, (**b**) comparison of predicted and experimental values of the training datasets and (**c**–**f**) comparison of experimental data, the prediction of the BPNN model and the θ projection model of the test datasets.

For MarM247LC superalloy, six creep curves, as indicated by black color in Figure 1, were selected for fitting the LM equation, which is P_LM_(*σ*) = *T*(C_LM_ + lg*t_f_*)10^−3^ = C_0,LM_ + C_1,LM_lg*σ* + C_2,LM_lg^2^*σ* + C_3,LM_lg^3^*σ* [16]. As seen in Figure 2a, the R^2^ value reaches 0.98, indicating the equation can be used to predict the *t_f_* under different conditions. Accordingly, the coefficients C_LM_, C_0,LM_, C_1,LM_, C_2,LM_, C_3,LM_ were determined to be 16.78, 53.1, −19.12, 2.76 and 0.002, respectively. With the fitted LM equation, *t_f_* under creep conditions of 900 °C/250 MPa, 950 °C/200 MPa, 950 °C/225 MPa and 1000 °C/125 MPa were determined to be 894 h, 427 h, 234 h and 962 h, respectively, as input parameters for the BPNN model. With six creep curves including 669 group data sets, the BPNN model was trained, as shown in Figure 2b. Apparently, the predicted data in the training datasets show good agreement with the experimental ones and the R^2^ reaches 0.99 in the iterative learning process. Subsequently, with the predicted *t_f_* values, stress, temperature and time, the creep strain under other creep conditions was predicted.

In the past decades, the θ projection model has been widely used to predict the creep behavior of various materials [17,18]. The multivariate linear relationship between the θ_i_ parameters and the creep conditions can be used to model the creep behavior of other stresses and temperatures. The specific relationship can be expressed as follows:

ε = θ_1_(1 − exp(−θ_2_t)) + θ_3_(exp(θ_4_t) − 1) (3)

where ε and t are the creep strain and creep time, respectively, and the parameters θ_i_ (i = 1, 2, 3, 4) are expressed a function of the creep conditions as follows:

logθ_i_ = a_i_ + b_i_*σ* + c_i_*T* + d_i_*σT* (4)

where *σ* is the creep stress, *T* is the creep temperature, and a_i_, b_i_, c_i_, d_i_ (i = 1, 2, 3, 4) are material constants. The θ_i_ values obtained by fitting the six creep curves are shown in Table 3. The material constants of MarM247LC superalloy and R^2^ are shown in Table 4. Then, the creep curves under any creep conditions can be predicted.

The comparisons between the prediction results of the BPNN model on the test datasets, the experimental results and the prediction of the θ projection model were shown in Figure 2c–f. As seen, the predicted creep strain of BPNN for all creep curves are close to the experimental ones, whose errors are within the range of ±20%. Moreover, it is obvious that the predicted data by BPNN were closer to the experimental ones than those predicted by the θ projection model for all conditions. The maximum error has been reduced by 30% with the BPNN model, compared with that by the θ projection model.

**Figure 3:**
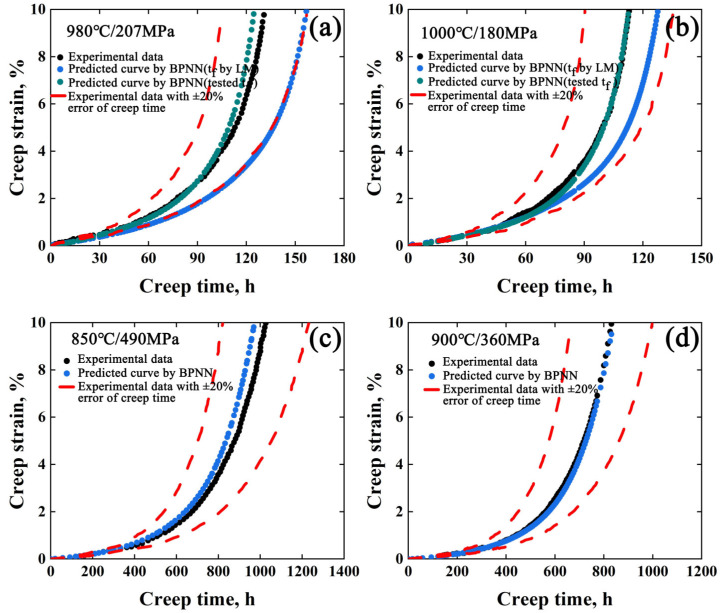
The results by BPNN model for DZ125 superalloy and CMSX-4 superalloy: (**a**,**b**) comparison of predicted and experimental values under 980 °C/207 MPa and 1000 °C/180 MPa for DZ125 superalloy, (**c**,**d**) comparison of predicted and experimental values under 850 °C/490 MPa and 900 °C/360 MPa for CMSX-4 superalloy.

On the other hand, the creep curves of DZ125 and CMSX-4 superalloys have been collected for the BPNN model with the same structure for training [12,13]. The creep curves of DZ125 superalloy under 900 °C/350 MPa, 950 °C/225 MPa, 950 °C/370 MPa, 980 °C/220 MPa, 1040 °C/137 MPa, and 1050 °C/102 MPa were trained and 980 °C/207 MPa and 1000 °C/180 MPa were tested. For CMSX-4 superalloy, the creep curves under 850 °C/430 MPa, 850 °C/560 MPa, 900 °C/400 MPa, 900 °C/460 MPa, 950 °C/250 MPa and 1000 °C/180 MPa were trained and 850 °C/490 MPa and 900 °C/360 MPa were tested. The predicted curves were compared with the experimental data for the two superalloys, as shown in Figure 3. As seen, the maximum prediction errors of the BPNN model are within ±20% for the two superalloys indicating that this method is suitable for different types of superalloys.

The prediction error for DZ125 superalloy under 980 °C/207 MPa and 1000 °C/180 MPa creep conditions are 20% and 15%, respectively, while the maximum error of creep curve prediction for CMSX-4 alloy is only ±5%, as shown in Figure 3c,d. It is apparent that the predicted data for CMSX-4 were closer to the experimental ones than those for DZ125 superalloy. To analyze the origin of errors, the predicted *t_f_* as input parameters were analyzed. The predicted *t_f_* of DZ125 alloy under 980 °C/207 MPa and 1000 °C/180 MPa are 174 h and 144 h, respectively, which are larger than the actual ones for 27% and 17%. Meanwhile, the predicted *t_f_* values of CMSX-4 alloy under 850 °C/490 MPa and 900 °C/360 MPa are 1125 h and 969 h, respectively, which are smaller than the actual ones for 5% and 1%. It is possible that predicted error is mainly incurred by the predicted *t_f_* values. To validify the above conclusion, the *t_f_* predicted by LM equation were replaced with the tested *t_f_*, and the previous trained model was used to predict creep curves, as seen in the green lines in Figure 3a,b. The results show that the maximum error of creep curve prediction is only ±4%. Therefore, it is necessary to predict *t_f_* accurately for the prediction of creep curves.

## 4. Conclusions

1. By using the BPNN model, the creep curves under different conditions were predicted. The maximum error of creep curves in the dataset is ±20%, which has been reduced by 30% compared with the θ projection model.
2. This method is applicable to the prediction of creep curves for other superalloys such as DZ125 and CMSX-4, and thus has a wide range of applications.
3. The accuracy of creep rupture life prediction plays an important role in the prediction accuracy of creep curves. Seeking the accurate prediction method for creep rupture life is of great significance for improving the predicted accuracy of creep curves.

## Figures and Tables

**Table 1:** The hidden layer and its corresponding mean square error (MSE) value.

Hidden Layer	MSE	Hidden Layer	MSE	Hidden Layer	MSE	Hidden Layer	MSE
2-1	5.30 × 10^−3^	5-2	8.83 × 10^−4^	32-2	6.25 × 10^−4^	16-8-4	1.09 × 10^−4^
5-1	5.63 × 10^−3^	8-2	7.86 × 10^−4^	8-8	2.68 × 10^−4^	16-8-2	6.20 × 10^−5^
16-1	5.64 × 10^−3^	16-2	5.79 × 10^−4^	16-8	3.83 × 10^−4^	16-8-8	5.68 × 10^−5^
20-1	6.37 × 10^−3^	20-2	8.32 × 10^−4^	16-6	2.40 × 10^−4^	16-8-10	5.93 × 10^−4^

**Table 2:** The parameters of the BPNN model in this work.

Type	Network
Hidden layers	3
Number of neurons	Input: 4(*T*, *σ*, *t*, *t_f_*)
	Hidden: 16-8-8
	Output: 1(*ε*)
Transfer function and training algorithm	Tanh(Xavier initialization) and Adam
Learning rate	0.001
Number of epochs	4000

**Table 3:** θ_i_ values obtained in MarM247LC superalloy by fitting the six creep curves corresponding to 0~10% creep strain range.

	900 °C/230 MPa	900 °C/300 MPa	900 °C/400 MPa	950 °C/150 MPa	950 °C/250 MPa	1000 °C/150 MPa
θ_1_	41,460	52,695	882,198	114,478	93,183	206,160
θ_2_	2.85 × 10^−8^	5.22 × 10^−8^	2.59 × 10^−8^	6.49 × 10^−9^	8.66 × 10^−8^	2.90 × 10^−8^
θ_3_	0.016	0.045	0.286	0.003	0.093	0.005
θ_4_	0.005	0.013	0.066	0.004	0.033	0.024

**Table 4:** Material constants corresponding to each θ_i_ parameter for MarM247LC superalloy.

θ Parameter	a_i_	b_i_	c_i_	d_i_	R^2^
θ_1_	−18.32	0.057	0.023	−5.37 × 10^−5^	0.97
θ_2_	6.30	−0.087	−0.015	9.75 × 10^−5^	0.77
θ_3_	10.58	−0.099	−0.016	1.19 × 10^−4^	0.99
θ_4_	−13.58	−0.010	0.011	1.90 × 10^−5^	0.99

## Data Availability

The data that support the findings of this study are available from the corresponding author, Jinshan He (email: hejinshan@ustb.edu.cn), upon reasonable request.
